# Developing and pre-testing a decision board to facilitate informed choice about delivery approach in uncomplicated pregnancy

**DOI:** 10.1186/1471-2393-9-50

**Published:** 2009-10-30

**Authors:** Jill Milne, Amiram Gafni, Diane Lu, Stephen Wood, Reg Sauve, Sue Ross

**Affiliations:** 1 School of Nursing, University of Victoria, British Columbia, Canada; 2 Department of Clinical Epidemiology and Biostatistics and the Centre for Health Economics and Policy Analysis, McMaster University, Hamilton, Canada; 3 Department of Family Medicine, Queen's University, Kingston, Canada; 4 Departments of Obstetrics and Gynaecology, University of Calgary, Calgary, Canada; 5 Departments of Community Health Sciences, University of Calgary, Calgary, Canada; 6 Departments of Paediatrics (Neonatology), University of Calgary, Calgary, Canada; 7 Department of Family Medicine, University of Calgary, Calgary, Canada

## Abstract

**Background:**

The rate of caesarean sections is increasing worldwide, yet medical literature informing women with uncomplicated pregnancies about relative risks and benefits of elective caesarean section (CS) compared with vaginal delivery (VD) remains scarce. A decision board may address this gap, providing systematic evidence-based information so that patients can more fully understand their treatment options. The objective of our study was to design and pre-test a decision board to guide clinical discussions and enhance informed decision-making related to delivery approach (CS or VD) in uncomplicated pregnancy.

**Methods:**

Development of the decision board involved two preliminary studies to determine women's preferred mode of risk presentation and a systematic literature review for the most comprehensive presentation of medical risks at the time (VD and CS). Forty women were recruited to pre-test the tool. Eligible subjects were of childbearing age (18-40 years) but were not pregnant in order to avoid raising the expectation among pregnant women that CS was a universally available birth option. Women selected their preferred delivery approach and completed the Decisional Conflict Scale to measure decisional uncertainty before and after reviewing the decision board. They also answered open-ended questions reflecting what they had learned, whether or not the information had helped them to choose between birth methods, and additional information that should be included. Descriptive statistics were used to analyse sample characteristics and women's choice of delivery approach pre/post decision board. Change in decisional conflict was measured using Wilcoxon's sign rank test for each of the three subscales.

**Results:**

The majority of women reported that they had learned something new (n = 37, 92%) and that the tool had helped them make a hypothetical choice between delivery approaches (n = 34, 85%). Women wanted more information about neonatal risks and personal experiences. Decisional uncertainty decreased (p < 0.001) and perceived effectiveness of decisions increased (p < 0.001) post-intervention.

**Conclusion:**

Non-pregnant women of childbearing age were positive about the decision board and stated their hypothetical delivery choices were informed by risk presentation, but wanted additional information about benefits and experiences. This study represents a preliminary but integral step towards ensuring women considering delivery approaches in uncomplicated pregnancies are fully informed.

## Background

The rate of deliveries by Caesarean section (CS) is increasing internationally [1-4]. Many factors have influenced this trend including changing clinical indications and maternal characteristics [5,6], as well as physician and maternal preferences. Physician preference for caesarean delivery has varied by country (UK 15-17% [7], Australia and New Zealand 11% [8], Ireland 7% [9]) and is generally attributed to fear of perineal trauma [8]. Maternal request for CS has also been widely documented [10-14]. According to a recent estimate between 4% and 11% of Caesarean deliveries worldwide are performed following maternal request in the absence of medical indication [15].

Concern about the rising rate of CS is based predominantly on an increase in maternal mortality and morbidity compared to vaginal delivery (VD), consequences for subsequent pregnancies and deliveries, neonatal respiratory morbidity [15], and cost implications [16]. In a recent study of over 100,000 pregnant women in eight Latin American countries [17] caesarean delivery was associated with a significantly increased risk of maternal morbidity for both intrapartum (OR 2.0, confidence interval 1.6 to 2.5) and elective cases (OR 2.3, confidence interval 1.7 to 3.1). Much of the on-going discussion has therefore focused on elective and 'on-demand' surgeries [18-21] and debates have been heated [22,23]; this is an emotive subject that touches on professional integrity, consumerism of patients, and ethics [24-26]. To address these concerns the UK National Institute for Clinical Excellence (NICE) released a detailed guideline which states that maternal request is not, on its own, an indication for CS [27]. The guideline emphasizes that clinicians should explore reasons for the request and discuss associated benefits and risks. A statement released by the Society of Obstetricians and Gynaecologists of Canada (SOGC) similarly confirms that the society 'does not promote Caesarean sections on demand,' but reiterates SOGC's commitment to patient choice [28]. The American College of Obstetricians and Gynecologists has recommended that while the principle of patient autonomy supports the right to undergo elective CS, the physician must ensure that each patient is fully informed regarding the risks and benefits of delivery options [29].

Despite a consistent international emphasis on informed decision-making, there remains a particular lack of clarity about how information related to delivery approach in uncomplicated pregnancies should be provided. This is concerning given the scarcity of medical literature about the relative risks and benefits of CS versus VD, and it is particularly unclear how to inform women about risks that are small [30]. The decision board may be an efficacious way to address this gap. Decision boards are decisional aids that provide a standardized base of written and graphic evidence-based information about treatment choices and associated risks/benefits [31]. The decision board is administered by care providers during consultations to support and guide rather than substitute for health-related counselling, thereby helping to ensure that both patient and care provider are informed about the broad range of factors that impact health-related decisions.

A number of trials have evaluated decision boards in varied clinical settings [32]. In their systematic review of 17 randomized trials, O'Connor and colleagues reported that decision aids were associated with higher knowledge scores and increased patient participation in decision-making, but had a variable effect on the decisions that were made [32]. In a study evaluating a decision board for women deciding between mastectomy and lumpectomy 91% of clinicians also reported that the tool had been a helpful part of the consultation [33].

The purpose of this article is to describe the development and pre-testing of a decision board that can be used in practice to guide discussion and promote informed and shared decision-making between health care providers and women who have the opportunity to choose between delivery options (VD or elective CS) in uncomplicated pregnancy. Before administering the decision board to pregnant women, we wished to determine whether women of child-bearing age who might face the decision in the future found the tool useful and informative, how the decision board would impact their decision-making, and what additional information they would want to receive before making a 'real' decision about method of delivery. Our overall aim was not to enter into the debate about the availability of on-demand CS but rather to develop a vehicle that communicates information in as unbiased a way as possible, and in a way that women can understand and use in discussion with their health care provider.

## Methods

### Development of the decision board

Development of the decision board comprised several phases. It was important to determine how best to present risks associated with CS and VD, since risks are generally small. We carried out two preliminary studies in which antenatal clinic patients (n = 40) were asked to imagine that they needed hernia repair surgery and were advised that associated risks were pain in the abdomen, further abdominal surgery, and/or hysterectomy. Outcomes included (1) preferences for presentation of risk-related information, and (2) which format (absolute risk, relative risk, or the Paling perspective, which visually presents risk on a logarithmic scale [34]) resulted in the most "correct" answers to the risk-based scenario. Correct answers involved the option with the least associated risk. Relative risk and the Paling perspective resulted in more correct answers (85% each) despite the fact that the majority of women (68%) reported that absolute risk was the easiest to understand. We therefore chose to represent risks numerically via absolute risk as well as graphically using the Paling perspective [34].

We also needed to select a source of information on which to base the presentation of medical risks associated with CS and VD. After reviewing the literature, we reached consensus that the NICE clinical guidelines [27] provided the most comprehensive review of medical risks at that time. Given the large number of risks included in the NICE guidelines, we undertook discussion within the research team (three team members work almost exclusively with pregnant women) to consider which data to include in the decision board. Priority was given to risks with higher probabilities, as well as those that addressed concerns that are commonly raised by patients. Seven items were chosen for inclusion: six items related to the mother (pain in the abdomen, death, bladder injury, infection, hysterectomy, leakage of urine) and one to the infant (breathing difficulty). After identifying the seven risks for inclusion, we developed the presentation of absolute risk and Paling perspective for each of the seven items.

**Figure 1:**
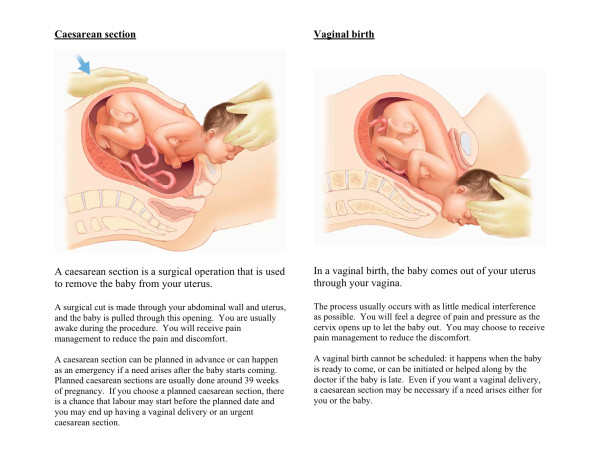
**Description of Caesarean section and vaginal birth**.

**Figure 2:**
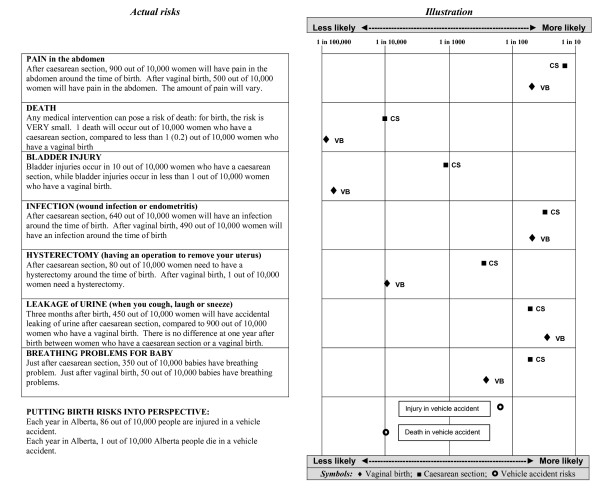
**Risk illustration**.

The last version of our decision board consisted of two sections. In Part One, graphic illustrations of each delivery approach (CS and VD) were combined with brief descriptions of the process involved (Figure 1). In Part Two, medical risks were presented numerically on the left as absolute risk and graphically on the right (Paling Perspective) in order that women could read across the page to obtain the same information in two formats (Figure 2). An obstetrician who was not involved with the study reviewed the decision board for potential bias towards VD or elective CS. The remainder of this article focuses on the pre-testing of this tool.

### Sample and recruitment

We chose to pre-test our decision board with women who were not pregnant because we did not wish to give the impression to pregnant women that CS was a universally available birth option. Therefore, women who were eligible for this study were of childbearing age (18-40) but were not pregnant. Since we were concerned that prior birth experience may have a major influence on women's decisions, eligible participants had also never given birth. A sample size of 40 was deemed appropriate for this initial study.

Following approval by the University of Calgary Conjoint Health Research Ethics Board, eligible women were identified from one family physician's office using the age-sex patient registry. Letters explaining the study and inviting participation were mailed from the physician's office. Women self-selected to take part by returning the signed consent form or calling the researcher (JM). As a trend towards highly educated participants became apparent, letters were mailed by a second family physician who served a more heterogeneous patient population.

### Data collection

Data were collected during individual interviews. Interviews were held in participants' homes or another location of their choosing and lasted approximately 30 minutes. Each woman was asked to imagine that she was pregnant, that she and her baby were healthy, and that she wished to discuss her delivery options with her physician. Participants recorded which approach to delivery they would choose (VD or CS) and completed a brief questionnaire, the Decisional Conflict Scale (DCS) [35] to evaluate how confident they were in their decision-making. The DCS addresses three aspects of decisional conflict: decisional uncertainty, factors contributing to that uncertainty, and perceived effectiveness of decision-making (Table 1) [36]. Responses to nine items, three in each subscale, are scored from 1 to 5, with the lowest scores representing the least uncertainty or most effective decision [35,36]. Although the choices made by the women in our study were in fact hypothetical, we wanted to examine how the use of the decision board would inform their decision-making.

**Table 1:** Items in decision conflict scale

**Informed Subscale**	**Uncertainty Subscale**	**Effective Decision Subscale**
1. I am aware of the choices I have in deciding on a method of birth	1. The decision is hard for me to make	1. I feel I have made an informed choice
2. I feel I know the benefits of caesarean section and vaginal birth	2. I am unsure which option to choose	2. My decision shows what is most important for me
3. I feel I know the risks and side effects of caesarean section and vaginal birth	3. It is clear which choice is best for me	3. I expect to stick by my decision

The researcher (JM) introduced the decision board and emphasized that the risks portrayed do not apply to all women in all circumstances. Each participant was informed that both CS and VD could involve unwanted consequences and that the medical risks were only some of the factors that might affect decision-making. The researcher also emphasized that despite planning an elective CS, labour could start before the planned date and/or an emergency CS could be required. Likewise, despite the intent to have a VD, an emergency CS could be necessary should the need arise for mother or baby. Participants read the decision board and were encouraged to comment and ask questions.

After reviewing the decision board, women again selected the birthing approach they would choose and completed the DCS. They then answered open-ended questions to evaluate the tool's perceived usefulness. The researcher reminded women that a major purpose of the study was to obtain critical feedback about the decision board, including whatever positive and negative comments they had. Conducting the interview after completing the DCS ensured that responses about decisional conflict were not influenced by the discussion. Three post-intervention open-ended questions asked women to consider (1) what they had learned, (2) whether or not the information had helped them to choose between birth methods, and (3) additional information that should be included. Another asked women to record factors that had influenced their choice of delivery method after reviewing the decision board. Women were prompted to take into account what they had read on the decision board (if relevant) as well as any factors external to the board, including personal values and experience. To ensure that women considered a wide range of factors, the final question asked women to review a series of potential factors and indicate (yes/no) which, if any, had influenced their decisions. Our intention was to stimulate discussion with study participants in order to inform future development of the decision board.

### Data analysis

Descriptive statistics were used to analyse sample characteristics and women's choice of delivery approach before and after exposure to the decision board. Content analysis was used to code and categorize narrative responses to open-ended questions as well as data from field notes. The first level of coding was used to identify the broad substantive area addressed by each unit of data. Two subsequent levels of coding identified emergent patterns and themes. Change in decisional conflict was measured using Wilcoxon's sign rank test for each of the three subscales.

## Results

Letters inviting participation in the study were mailed to 90 women from two family practices. Forty women, aged 20 to 40 (median age 27 years) agreed to participate. None had delivered a baby and none, to the best of their knowledge, were pregnant at the time of the study. Despite efforts to ensure varied educational levels most women (n = 36, 90%) had completed post-secondary education.

### Delivery approach and decisional conflict

Most women (n = 35, 87%) reported they would choose VD over elective CS prior to reading the decision board. This number increased to 37 (92%) post-intervention when two women who had previously been undecided chose VD (Table 2). Three women (8%) opted for CS pre and post-decision board. Although their choice of birth method remained generally unchanged, women were more confident in the decision they had made at the end of their interviews. There was a significant decrease in decisional uncertainty (p = 0.001) and factors contributing to that uncertainty (p < 0.001) after reviewing the decision board. There was also a significant increase in the perceived effectiveness of decisions (p < 0.001).

**Table 2:** Choice of delivery method

**Choice of delivery method**	**Choice pre-decision board**	**Choice post-decision board**
	**(n = 40)**	**(n = 40)**
**Vaginal birth**	35 (87%)	37 (92%)
**Caesarean section**	3 (8%)	3 (8%)
**Undecided**	2 (5%)	0

### Perceptions of the decision board

The majority of women commented positively about the decision board. Most reported that the decision board had helped them choose between birth methods (n = 34, 85%), and had generally reinforced their decisions to have a VD. Comments included:

*It informed me of the actual risks so I know that I made an informed decision*.

*The side-by-side comparison helped to bring things into perspective*.

*It clearly showed the difference in outcome if you choose a vaginal delivery or caesarean section*.

The majority of women believed that the decision board would be useful in their decision-making if they were pregnant (n = 32, 80%).

### New information

Most women thought they had learned something new from the decision board (n = 37, 92%). Seven had learned about the risks associated with both birth methods. Only one woman (who would opt for CS) believed that risks were "almost in line with each other;" the majority reported that risks were higher for CS than VD. Comments included:

*Before I knew that vaginal birth was safer but I didn't know the actual effect of injury and the effect on the baby*.

*I learned that the risks of caesarean section are higher than vaginal delivery. I thought it was the other way around*.

Women also commented on specific risks. Fifteen were "surprised" about the increased risk of hysterectomy associated with CS; one stated this was the most powerful factor on the list and would deter her from undergoing an elective CS. Seven women said the risk of bladder injury was new to them and five commented on the potential for breathing problems in the CS baby. Fewer remarked about the risks of infection, urinary leakage, pain, and death.

### Factors impacting choice of delivery method

One open-ended question asked women to record factors (personal and/or risk-related) that had influenced their post decision board choice of delivery approach. Most women (75%) who chose VD emphasized the importance of risk factors in general. Specific risks that stood out were hysterectomy, bladder injury, and infection. For many (n = 13) VD was the "normal" or "natural way to go"; "the way that it's meant to happen." The desire to avoid surgery (n = 12) and the longer recovery time associated with CS (n = 5) were additional factors cited by women who chose VD. Eight had been influenced by friends and family who had delivered vaginally.

All three women who would elect to have a CS cited first hand experiences as factors in their decision-making. Two noted that they and their siblings were delivered by CS; one admitted that the thought of VD scared her and the other perceived CS as a "less stressful and embarrassing, and a simpler option." The third reported that her professional awareness of the "implications on the infant when VD goes wrong", as well as the importance of timing/preparation, had influenced her decision.

Responses to the yes-no statements addressing relevant factors (Table 3) generally supported the impact of risk-based knowledge related to maternal health (75%) and health of the baby (70%). Specific factors reported to be influential by more than 25% of participants included breathing problems for the baby, pain after birth, bladder injury, hysterectomy, and the potential need for additional surgery.

**Table 3:** Responses to list of factors impacting decisions regarding delivery method

**Factor**	**n**	**%**
Knowledge of risks for myself	30	75%
Knowledge of risks for baby	28	70%
Worry about impact on baby	21	52.5%
Wanting more children	20	50%
Wanting to fully experience birth	17	42.5%
Wanting to have options in births of future babies	17	42.5%
Worry that baby may have breathing problems	16	40%
Worry about pain after birth	16	40%
Wanting to bond with baby	14	35%
Worry that bladder may be injured	11	27.5%
Worry re hysterectomy	11	27.5%
Worry I might need surgery after birth	10	25%
Worry re pain during birth	9	22.5%
Worry re pain in abdomen after birth	9	22.5%
Concern re ability to breast feed	8	20%
Desire to avoid scar	8	20%
Wanting to avoid UI	8	20%
Concern I might die	7	17.5%
Needing to plan date with family	5	12.5%
Being uncertain re ability to cope with childbirth	5	12.5%
Wanting to plan date with doctor	4	10%
Wishing to avoid labour pains	3	7.5%
Wishing to avoid stretch marks	1	2.5%
Took no factors into account	0	

### Missing information

Women commonly reported that risks to the baby and benefits of VD over CS had been under-represented. Several also noted that risks were predominantly associated with delivery and did not address the recovery period and onwards. Others wanted more detail about the pain associated with VD and CS, that is, what they would experience and when, and options for pain management. Several women said they wanted to know more about the actual experiences of VD and CS.

In terms of specific risks, women wanted more explanation about bladder injury and infection. For example, one asked about the meaning of bladder injury and what type of infection can be associated with VD. Another wanted clarification on what "around the time of" meant in relation to risks such as infection. These two women thought that the decision board raised more questions than it answered.

## Discussion

Our study is the first that we are aware of to develop and examine a decision aid to facilitate decision-making between elective CS and VD in women with uncomplicated pregnancies. Women of childbearing age who were not pregnant and had never given birth responded positively. Most thought that they had learned something new and that risk-related information would impact their decision-making regarding delivery approach. Their decisions changed little after using the decision board but women were more confident in their choices, stating that the process confirmed and added to the information they had pre-intervention. The enhanced confidence was most evident in two women who were initially undecided but chose VD after reviewing the decision board, citing risk factors associated with CS.

Our findings are similar to those reported in trials that have evaluated decision aids in a variety of clinical settings: decision aids improved knowledge and decreased decisional conflict [32]. Decision aids for women choosing delivery approach following a previous CS have been evaluated in randomised trials [36,37]. An Australian trial examined a decision aid booklet, which included risks and benefits of each approach [36]. Women randomised to receive the booklet at 28 weeks gestation (n = 115) demonstrated a significant increase in focused knowledge scores (p < 0.001) and a significant decrease in decisional conflict (p < 0.05) compared with women in the control group (n = 112). Although preference for trial of labour was similar between groups at 36 weeks, women in the intervention group were less likely to be "unsure" of their decision [36]. The second, larger UK trial [37] compared usual practice with two forms of decision aid: (1) a computer-based information program relating possible health outcomes and probabilities associated with VD, elective CS and emergency CS, and (2) a separate program that asked women to value possible health outcomes using a visual analogue scale, but omitted probabilities. Women in both intervention groups had higher knowledge scores and lower decisional conflict than those in usual care, and although there was no statistical difference in mode of delivery between groups, researchers postulated that the higher rate of vaginal birth in the group that attached values to the outcomes was clinically important. Another trial evaluating a decision aid (consisting of a booklet plus an audio-CD and worksheet) about management options for women with breech presentation at term also found that the decision aid improved knowledge and reduced decisional conflict [38], although there was no statistically significant difference between groups in the proportion of women undergoing external cephalic version.

Our study contributes to this expanding body of knowledge by addressing choice of delivery approach in women who have not had a prior CS and who have no known complications, that is, those women who may seek a CS 'on demand'. Participants reported a significant decrease in decisional conflict and a significant increase in the perceived effectiveness of their decisions after reviewing the decision board (p = 0.001). The interpretation of these findings, however, merits caution. Our sample size was small (n = 40) and most women were highly educated. Participants were representative of an academic GP's practice (DL) but may not be representative of the larger population of women in our city between the ages of 18 and 40. Moreover, since on-demand CS is not currently a universal option we wished to avoid misleading pregnant women by raising the expectation that CS would be available to all of them. We therefore chose to pre-test the decision board with non-pregnant women to obtain feedback related to the tool's comprehensiveness and feasibility. To enhance the transferability of our findings we recruited participants of childbearing age. These women were excellent critics of the decision aid, were vocal in their praise and criticism of the decision board, and voiced numerous beliefs/perceptions that have been previously reported by pregnant women [38-40].

Despite potential criticism about the simplistic descriptions we used of the interventions and limited number of possible risks associated with the interventions, our findings support the usefulness and feasibility of the decision board approach and constitute an integral step in our overall aim to develop a vehicle for communicating risk-based information related to delivery approach (VD, elective CS). Women appreciated the systematic presentation of information and complementary ways of formatting the data. They provided equally important feedback, however, about information that they thought had been omitted from the decision board, including risks to the baby, realistic detail about the actual experience of undergoing VD and CS, the associated pain and pain management options, the risk of perineal tears and/or episiotomy with VD, and the recovery period following both VD and CS. This feedback will be critical to future revisions of our decision board, and women's suggestions will be incorporated to reflect the most current evidence as we work to create and evaluate a more comprehensive tool [41]. Additional detail will more accurately reflect what is meant by bladder injury and infection, while pain control options associated with VD and CS will be more comprehensively described. Attention will be also paid to the descriptions of delivery approaches in Figure 1, which several women believed were overly simplified, and to the textual descriptions of VD and CS which may have biased the decision-making process [42]. Perhaps most importantly, potential sequelae of choices that are made will be added to guide discussion with health care providers, and to ensure that women understand that attempted VD may result in significant medical interventions (such as labour induction, epidural), instrumental delivery (forceps or vacuum) or emergency CS. Current research suggests that the risk of emergency CS is less than 5% for multiparous women in spontaneous labour at term but may be as high as 35% for primiparous women who undergo induced labour at 42 weeks gestation [43]. Augmentations to the decision board will promote understanding that the health and safety of mother and baby will ultimately dictate delivery approach.

In the next phase of this research program it will be important to evaluate the revised decision board with care providers and women who are pregnant. Pregnant women are likely more motivated to seek focussed information and may therefore differ in their views of what is relevant, useful, and important. Repeated series designs should inform the impact of the decision board at varied intervals throughout pregnancy, while focus group interviews will provide a means to help ensure that content is both comprehensive and relevant given the varied circumstances and needs of pregnant women. As decision aids are meant to support medical consultation, the decision board will need to be tested in clinical practice to incorporate the two-way discussion that is critical to shared patient/health care provider decision-making [30].

As debate continues about the ethics of offering on-demand CS, the question for health care providers remains how to best counsel patients who request elective CS. At the time we were developing the decision board, the evidence presented in the NICE guidelines [27] represented the most rigorously and independently evaluated risks of CS and VD. Clearly, however, there is much work still to be done including the need for well-designed studies comparing short and long-term outcomes of VD and elective CS [22]. As a dynamic tool reflecting the current state of evidence and the needs of stakeholders (health care professionals and their patients), a decision board may best be described as a work in progress.

## Conclusion

While clearly a controversial issue, the choice to undergo elective CS is being exercised to varying degrees by women in Canada and internationally [15,17,44-47]. This study represents a preliminary but integral step to help ensure women considering delivery approaches in uncomplicated pregnancies are fully informed. Our decision board affords a standardized means to organize data that non-pregnant women found useful and future iterations based upon our findings may guide discussions between patients and health care providers.

## Competing interests

The authors declare that they have no competing interests.

## Authors' contributions

JM contributed to the design of the study, interviewed participants, played a major role in the analysis and interpretation of data, and drafted the manuscript. AG played a major role in the conception and design of the study, data analysis, and the drafting and revising of the manuscript. DL played a major role in the conception and design of the study and contributed to all stages of manuscript preparation and revising. SW and RS contributed to the design of the study and manuscript preparation/revising. SR conceived of the study, played a major role in its design and in all stages of data collection and analysis, and in the preparation and revising of the manuscript. All authors have read and given final approval of the manuscript as submitted.

## Pre-publication history

The pre-publication history for this paper can be accessed here:


